# CircPrimer: a software for annotating circRNAs and determining the specificity of circRNA primers

**DOI:** 10.1186/s12859-018-2304-1

**Published:** 2018-08-03

**Authors:** Shanliang Zhong, Jinyan Wang, Qian Zhang, Hanzi Xu, Jifeng Feng

**Affiliations:** 1 Center of Clinical Laboratory Science, Jiangsu Cancer Hospital & Jiangsu Institute of Cancer Research & The Affiliated Cancer Hospital of Nanjing Medical University, Nanjing, 210009 China; 2 Department of General Surgery, The Affiliated Cancer Hospital of Nanjing Medical University, Nanjing, 210009 China; 3 Department of Radiation Oncology, Jiangsu Cancer Hospital & Jiangsu Institute of Cancer Research & The Affiliated Cancer Hospital of Nanjing Medical University, Nanjing, 210009 China; 4 Department of Medical Oncology, Jiangsu Cancer Hospital & Jiangsu Institute of Cancer Research & The Affiliated Cancer Hospital of Nanjing Medical University, Baiziting 42, Nanjing, 210009 China

**Keywords:** Circular RNAs, Divergent primer, Sequence

## Abstract

**Background:**

Since circular RNAs (circRNAs) post-transcriptionally regulate gene expression, they have attracted increasing attention. However, there is no existing tool to annotate and extract spliced sequences for circRNAs and no tool to determine the specificity of circRNA primers.

**Results:**

In this study, we present circPrimer, which allows users to search, annotate, and visualize circRNAs. Additionally, circPrimer enables users to extract the spliced sequences and genomic sequences of any circRNA, including novel circRNAs. Furthermore, circPrimer help users to design primers for circRNAs and to determine the specificity of the circRNA primers.

**Conclusions:**

CircPrimer is a user-friendly tool for exploring circRNAs that does not require special user skills.

## Background

Circular RNAs (circRNAs) are a large class of regulatory RNAs that were identified in the early 1990s, and in the following years, they were considered to be molecular flukes or products of aberrant RNA splicing [1, 2]. Recently, with advances in high-throughput RNA sequencing (RNA-seq) technology, circRNAs were revealed to post-transcriptionally regulate gene expression and have gained increasing attention [2]. CircRNAs may exert their functions by serving as miRNA sponges [3–5], binding proteins [6], coding proteins [7, 8], modulating the transcriptional activity of RNA Pol II [9], and competing with linear splicing [10]. CircRNAs also serve as potential biomarkers for cancer detection and therapy [11–13].

To identify circRNAs from high-throughput RNA-seq data, a number of tools have been developed. In 2012, Salzman et al. developed a computational method to look for circRNAs in RNA-seq datasets [14]. Next, Find_circ [15], MapSplice2 [16], Segemehl [17], circExplorer [18], circRNA_finder [19], CIRI [20], ACFS [21], KNIFE [22], NCLscan [23], DCC [24] and UROBORUS [25] were developed in succession. With the increasing number of circRNAs identified using these tools, several databases have been established to collect and organize the circRNA sequences and information. For example, CircBase merged and unified several data sets of circRNAs into a standardized database, where investigators can search, browse and download genomic annotations of circRNAs [26]. CSCD (Cancer-specific circRNAs database), a database developed for cancer-specific circRNAs, collected the available RNA-seq datasets from 87 cancer cell line samples [27]. However, there is no existing tool to annotate and extract spliced sequences for circRNAs. Since circRNAs derived from the same parental gene may share the same sequence, divergent primers for one of these circRNAs may also amplify others. Furthermore, a previously reported tool named CircInteractome [28] was unable to generate a template for a novel circRNA or design primers with one primer spanning the spliced junction. To date, no tools have checked the specificity of circRNA primers. In present study, we introduced a tool that not only searched and annotated circRNAs but also helped users to design primers and determinthe specificity of the primers.

## Implementation

CircPrimer is written in delphi. The circRNAs from *Homo sapiens* were downloaded from circBase and were imported into a local database. RefSeq GTF files, hg19 and hg38, were downloaded from UCSC (http://genome.ucsc.edu/) [29]. The RefSeq GTF files, GRCh37.75 and GRCh38.90, were downloaded from Ensemble (ftp://ftp.ensembl.org/pub) [30]. To annotate a circRNA, we first searched the RefSeq GTF files for transcripts with a genomic location containing the genomic location of the circRNA. Next, the transcripts were scored as following: If the start position of the circRNA exactly matched the 5′ end of an exon in the transcript, 3 points were assigned; If the end position of the circRNA exactly matched the 3′ end of an exon, another 3 points were assigned. If one boundary of the circRNA was located on an exon but did not exactly match the end of this exon, 2 points were assigned (2 points for each boundary); otherwise, 0 point was assigned. The transcript with highest sum score was extracted and defined as the parental gene of this circRNA. If two or more transcripts were obtained, the longest one was extracted.

Since Gao et al. suggested that only 2.7–4.3% circRNAs were alternatively spliced [31], we used the following method to predict the spliced sequences of the circRNAs. We first extracted the genomic sequence from UCSC according to the genomic location of the circRNA. Next, the circRNA was annotated using the method described above. According to the annotated result, the intron sequences were removed from the genomic sequence of the circRNA. Nevertheless, if one or two boundaries of the circRNA were located in an intron, the intron sequence from the start position of this circRNA to the first exon or the intron sequence from the last exon to the end position was retained.

To design primers for circRNAs, sequences can easily be searched and obtained. The template can be generated for primer3 [32] to design divergent primers or primers with one primer spanning the spliced junction.

To check the primer specificity, sequences in the circBase were searched to show that the potential circRNAs could be amplified by the primers. The position of the primer on the circRNA can also be shown visually.

## Results

### Data input

The CircRNA ID (e.g., hsa_circ_0000007), genomic location (e.g., chr1:1735857–1,737,977), gene symbol, or a file path of the text file (one genomic location per line) can be input into the circRNA field, depending on the function that the users will use. To input a file path, users press the Ctrl key and later double click the circRNA field to show the open file dialog. The chromosome, the start and end coordinates, and the strand orientation can be separated with any non-numeric character, except ‘,’.

### Searching circRNAs in circBase

**Fig. 1:**
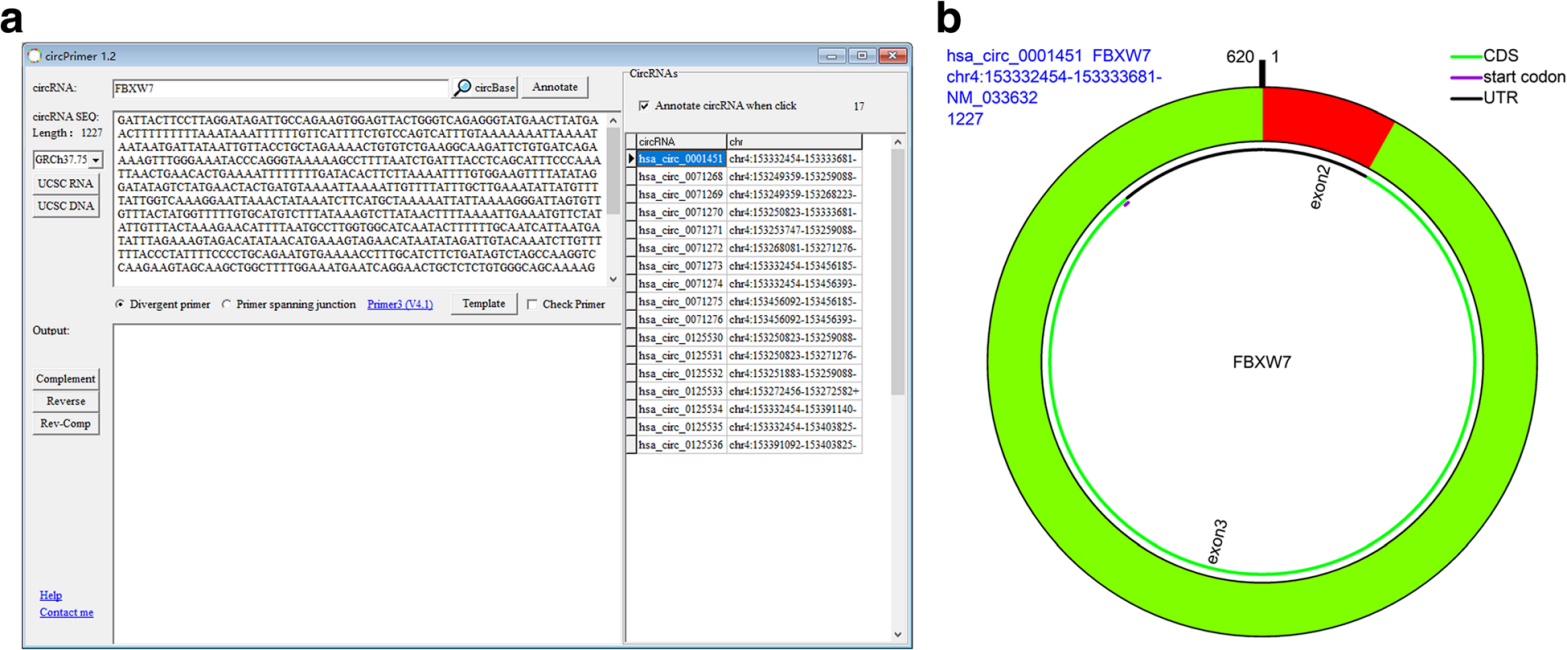
Searching and annotating circRNA using circPrimer. **a** Search results for gene symbol, FBXW7; **b** Annotation result for has_circ_0001451. CDS, coding sequence; UTR, untranslated region

The CircRNA ID, genomic location, and gene symbol are the accepted data for searching circRNAs in the circBase. After inputting the data into the circRNA field and clicking the “circBase” button, the circRNAs are listed at the right of the main form (Fig. 1a), if one or more circRNAs are obtained. When clicking one of the listed circRNAs, the sequence of this circRNA is shown in the field ‘circRNA SEQ.’ If ‘Annotate circRNA when click’ is checked, a form is presented to show the annotated result of this circRNA (Fig. 1b). It should be noted is that RefGene ‘GRCh37.75’ or ‘hg19’ should be chosen before clicking a circRNA to annotate the circRNA because the circRNAs in circBase were mapped to the hg19 human genome. Users can save the search results in fasta format by using the right-click list menu.

### Annotating a circRNA

The CircRNA ID and genomic location are the accepted data for annotating a circRNA. After inputting the data to the circRNA field and clicking the ‘Annotate’ button, a form is presented to show the annotated result of the circRNA. The data presented in the figure are extracted from circBase or are annotated according to the selected RefSeq GTF file. To distinguish the different data sources, the blue text indicates the information from circBase, and the black text indicates the annotated results. There are two reasons for when there is difference between the data from circBase and the annotated results. The first reason is that the user did not select the right refGene version. As mentioned above, RefGene ‘GRCh37.75’ or ‘hg19’ should be chosen when annotating a circRNA in circBase. The other reason is that there might be a mistake in the circBase data or our annotated results. For example, Yang et al. reported that [33] the sequence and the length of the sequence of hsa_circ_0001451 were different from those in circBase. After annotating hsa_circ_0001451 using circPrimer, we found that circPrimer obtained the same sequence that Yang et al. reported, and the sequence in the circBase was the genomic sequence of hsa_circ_0001451. Therefore, when users find a difference, they should confirm the sequence using an experimental method. When the genomic location of a novel circRNA is inputted, all of the presented data are annotated by circPrimer, and only the genomic location, gene symbol and sequence length are presented on the top-left of the figure.

### Extracting a spliced sequence

The CircRNA ID, chromosome location, and a file path are the accepted data. After inputting the circRNA ID or chromosome location and later clicking the ‘UCSC RNA’ button, the spliced sequence of the circRNA is shown in the ‘circRNA SEQ’ field. When inputting a file path for a file with multiple chromosome locations, a circRNA list will be presented at the right of the main form. Users can annotate a circRNA or export the circRNAs in fasta format using the method mentioned above. It should be noted that no matter which data is input, the sequence is not obtained from circBase but is extracted from the UCSC web site according to the annotated results.

### Extracting the genomic sequence

The CircRNA ID, chromosome location, and a file path are the accepted data. After inputting the circRNA ID or chromosome location and subsequently clicking the “UCSC DNA” button, the genomic sequence of the circRNA is shown in the “circRNA SEQ” field. If you input a file path of a file with multiple chromosome locations, a text file in fasta format will be saved in the root directory of the application.

### Designing and checking primers

Designing specific primers for the quantification of circRNAs is challenging and prone to errors, since the mature circRNA sequences after splicing are not readily available in many cases, and the primers must be divergent and must span the junction.

Users can easily obtain the spliced sequence of a circRNA using circPrimer, and it does not matter of the circRNA is a novel or already known. After obtaining the spliced sequence, depending on the users’ selection, circPrimer generates a template for designing divergent primers or primers with one primer spanning the spliced junction. Users just paste the generated template into Primer3 to design primers for polymerase chain reaction (PCR). To check the specificity of the circRNA primers, users check “Check Primer” to show the panel “Check Primer” and the input the primers and, finally, click the “check” button to start the checking process.

**Fig. 2:**
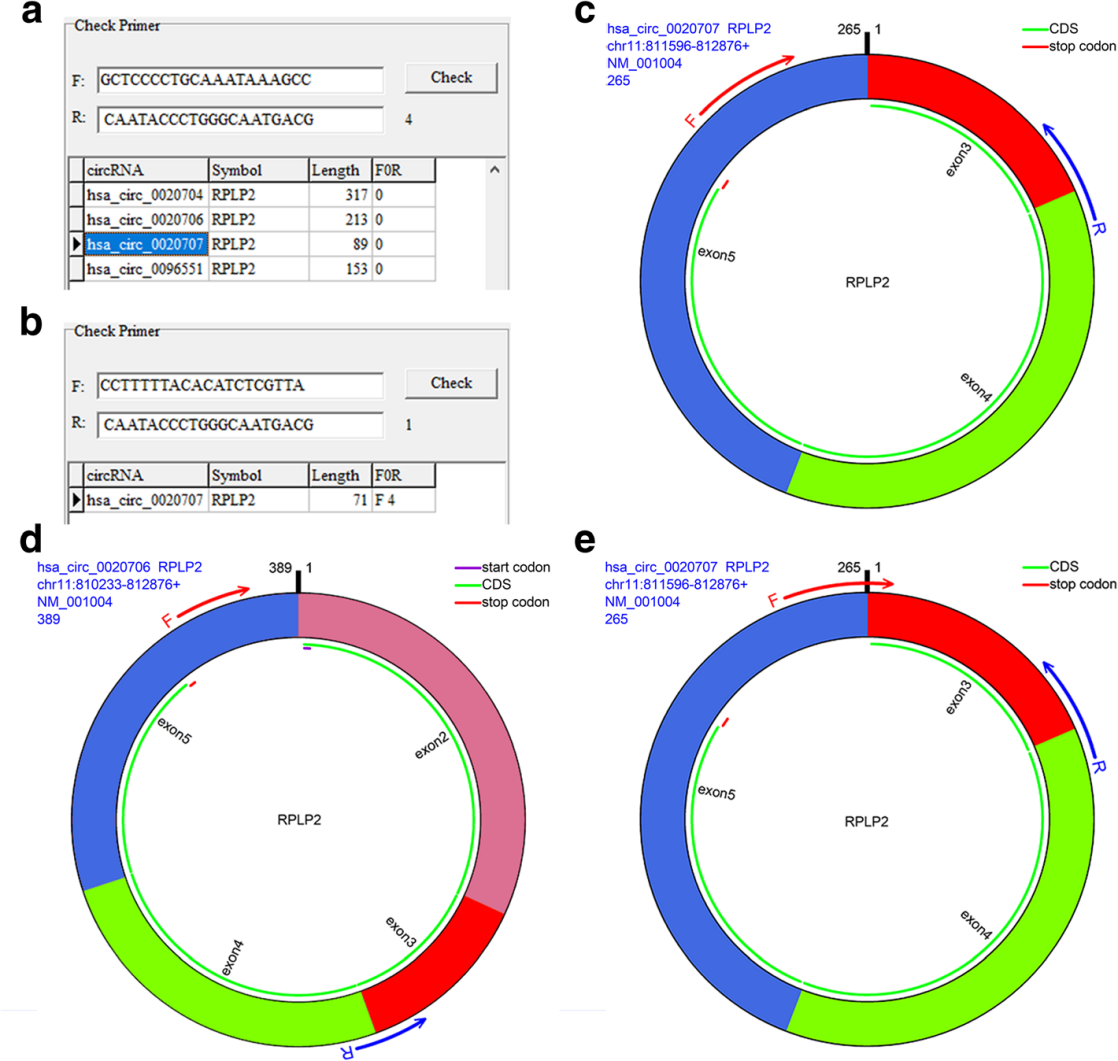
Checking the specificity of circRNA primers. **a** Checking the specificity of divergent primers for has_circ_0020707, a circRNA derived from RPLP2. Length, polymerase chain reaction (PCR) product size; F0R, primer characteristic (1, convergent primer; 0, divergent primer; F + No., forward primer spanning the spliced junction [No. represents the base count spanned by the primer]; and R + No., reverse primer spanning the spliced junction). **b** Checking the specificity of primers, with one primer spanning the spliced junction. **c** Localization of the divergent primers in has_circ_0020707. **d** Localization of the divergent primers in has_circ_0020706, which is also derived from RPLP2. **e** Localization of the primers, with the forward primer spanning the spliced junction of has_circ_0020707

The reason for designing the primers with one primer spanning the splicing junction is that the divergent primer may not be specific enough to amplify the target circRNA. As shown in Fig. 2a, the divergent primers designed for hsa_circ_0020707 amplify 4 circRNAs, which are derived from same parental gene (i.e., RPLP2) and share the same sequence (Fig. 2c and d). If one of the primers spans the spliced junction of the circRNA, the amplification could be more specific than the divergent primers (Fig. 2b, c and e). However, when five or more bases located in the 3′ end of a primer span the spliced junction, these types of primers have the possibility to amplify the parental gene of the circRNA.

At the present time, there is no tool to directly design primers for circRNA. The previously reported tool, CircInteractome, also used Primer3 or the NCBI Primer Design tool to design primers for circRNA [28]. However, CircInteractome is unable to generate a template for a novel circRNA or to check the specificity of the primers.

## Conclusions

We developed a user-friendly tool to annotate circRNAs that does not require any special user skills. With this tool, users can easily search circRNAs and annotate circRNAs visually. Users can extract the spliced sequences and genomic sequences of any circRNA, including novel circRNAs. Furthermore, circPrimer helps users to design primers for circRNAs and to determine the specificity of the circRNA primers.

## Availability and requirements

Project name: circPrimer

Project home page: http://www.bioinf.com.cn/

Operating system(s): Window

Programming language: Delphi

Other requirements: Internet connectivity

License: GNU General Public License version 3.0 (GPL-3.0)

Any restrictions to use by non-academics: None

## Abbreviations

circRNA: Circular RNA
CSCD: Cancer-specific circRNAs database
RNA-seq: RNA sequencing
